# Green finance and corporate environmental investment: "Scale Up" or "Efficiency Up"?

**DOI:** 10.1371/journal.pone.0297456

**Published:** 2024-02-12

**Authors:** Qu Yang, Shiyi Ming, Rongguang Zhang, Haitao Yan

**Affiliations:** 1 Business School, Chengdu University of Technology, Chengdu, 610059, China; 2 Business School, Sichuan Normal University, Chengdu, 610066, China; Universiti Malaysia Sabah, MALAYSIA

## Abstract

The establishment of green finance reform and innovation (GFRI) pilot zone is an important measure of the Chinese government to urge enterprises to develop green transformation. This paper explores the impact of pilot policies in the GFRI pilot zone on corporate environmental investment. Based on 819 A-share listed enterprises from 2010 to 2020, our staggered difference-in-differences (staggered DID) estimation documents revealed that enterprises in the GFRI pilot zone significantly increased the corporate environmental investment efficiency but reduced the scale of corporate environmental investment.This conclusion remained robust after Propensity Scores Matching difference-in-differences (PSM-DID), replacing dependent variables, and shortening the time window. We contend that the increased research and development (R&D) expenditure and technological innovation are the potential mechanisms at work. Heterogeneity analysis showed that the establishment of GFRI improved the environmental investment efficiency of polluting enterprises but had no effect on green enterprises.Meanwhile, the effect of GFRI exhibited heterogeneity in the type of enterprise ownership. This paper evaluates the implementation effect of GFRI from the perspective of corporate environmental investment, and provides theoretical support and an empirical basis for green finance policy to serve China’s green economy.

## 1. Introduction

China has maintained a relatively high economic growth rate in recent years. However, some problems have been associated with this process, including tighter resource constraints, serious environmental pollution, and ecosystem degradation. China has also become the world’s largest carbon emitter [1]. In the future, the country will face increasingly severe resource constraints and huge ecological restoration costs [2]. At present, the world has reached a consensus on developing low-carbon economy to promote sustainable development. How to achieve sustainable development under the background of low-carbon economy is a common problem facing the world today [3,4]. To achieve sustainable development, China’s economic development model requires both speed and quality, i.e., maintaining economic growth while avoiding over-exploitation of the environment and resources [5,6]. Therefore, the Chinese government has proposed policies such as low-carbon cities and green finance that take into account both economic development and environmental protection. Among them, green finance has financial attributes and environmental benefits, which can effectively realize low-carbon economic development [7]. As a financial innovation, green finance mainly addresses the problems of enterprise energy conservation and green project financing, which is essentially credit rationing based on environmental constraints. It stems from the “Equator Principles” proposed in 2002, which require financial institutions to consider corporate environmental performance into account in credit funding [8]. The environmental pollution problem of enterprises will be transformed into the financing cost of enterprises, which will increase the financing cost of highly polluting industries and reduce their return on investment [9,10]. In 2007 the European Investment Bank issued its first green bonds, the proceeds of which were used to finance warming mitigation projects. Subsequently, the Paris Agreement was signed to give a strong boost to the international carbon market. It brought an abundance of global low-carbon investments. To achieve the Sustainable Development Goals, the concept of green finance has been introduced and is rapidly developing in China [11–13]. In 2017, China decided to establish the first batch of Green Finance Reform and Innovation (GFRI) pilot zones in Guangdong, Zhejiang, Jiangxi, Guizhou, and Xinjiang provinces, to promote the development of green finance and guide the allocation of financial resources to green environmental protection projects. Gansu Province and Chongqing Municipality were added to the GFRI pilot zones in 2019 and 2022, respectively, to enrich the practical experience in GFRI.

The main tasks of the GFRI pilot zone are as follows: supporting financial institutions to increase credit funds for green projects, such as pollution control and emission reduction; encouraging financial institutions to take environmental protection and resource conservation as an important basis when making credit decisions and developing financing standards; and subsidising enterprises’ green technology innovation [14,15]. In the GFRI pilot zone, enterprises can use green projects or green technologies as vouchers for financing, and at the same time, enterprises can obtain "reputation effect" [16,17]. Financial institutions use this as a standard to help enterprises obtain financing. Firms are able to use financing to develop technology and update equipment [18]. Therefore, GFRI will ensure that firms consider their own environmental investment when financing, thereby affecting their environmental investment strategy. Enterprises are the main producers of carbon emissions and consumers of energy. Enterprises wanting to obtain increased credit funding will certainly optimise their corporate environmental investment and fulfil their environmental/social responsibility [19]. On the other hand, it is difficult for enterprises with high pollution and high emissions to be favoured by financial institutions [20]. However, the impact of green finance reform on corporate environmental investment strategies still needs to be explored. On the positive side, Huang et al. (2021) [21] reported that green investment is conducive to regional green technology innovation to achieve the regional carbon emission reduction goal. Green credit policies also encourage the expansion and development of green enterprises and promote the development of green technology by environmental protection enterprises [22,23]. At the same time, the excellent environmental responsibility performance of enterprises will serve as an important guarantee for creditors to evaluate their credit risk. On the negative side, Wang et al. (2019) [24] found that green credit policies did not help high-polluting enterprises to transform technologically or upgrade their industrial structure, nor did such policies become a decision-making tool for risk management in financial institutions. Specifically, the following questions remain unclear: (1) Does GFRI change corporate environmental investment? (2) Does GFRI alter the scale or efficiency of corporate environmental investment, or does it improve both of these aspects? At present, there is a lack of reliable empirical evidence.

This paper utilised the data of 819 A-share listed companies from 2010 to 2020 as samples. We adopted the staggered DID method to evaluate the impact, internal mechanism, and heterogeneous effect of GFRI on corporate environmental investment. We observe that that establishing GFRI improved the efficiency of corporate environmental investment, while the scale of corporate environmental investment decreased. Most enterprises choose "improving efficiency" to obtain the support of green finance pilot policies. After selecting different matching methods, replacing explained variables, and shortening the time window, our conclusion remained robust. Further, we also found that GFRI pilot area can increase enterprises’ R&D investment and enhance their technological innovation ability, thus improving the efficiency of corporate environmental investment. Therefore, enterprises in the GFRI pilot area can reduce unnecessary environmental expenditure. They will allocate limited internal resources to R&D investment to improve technological innovation, and ultimately achieve the goal of green development.Their actions also confirm the resource-based view that firms have limited internal resources [25]. Through heterogeneity analysis, we observed that most enterprises could improve their technological innovation and environmental investment efficiency by reducing their environmental investment to increase R&D investment.

The contribution of our paper is twofold. First, we enriched the literature on the economic consequences of green finance and expand the theoretical research on the driving factors of corporate environmental investment. The existing literature mainly discusses how green finance affects technological innovation [26,27]and green total factor productivity [28]. Based on the consideration of technological innovation and R&D expenditure, we divided corporate environmental investment into two aspects to study the impact of GFRI on corporate environmental investment, we divided corporate environmental investment into two aspects: corporate environmental investment scale and corporate environmental investment efficiency. Secondly, we enriched the research on corporate environmental investment. Ma et al. (2016) [29] found that environmental regulations increase enterprises’ spending on environmental protection. However, Zhao et al. (2022) [30] believed that more advanced environmental protection technologies should be learned by increasing R&D investment to enhance the efficiency of environmental protection investment. Therefore, according to the ideas in the existing literature, we identified two factors affecting GFRI corporate environmental investment: R&D expenditure and innovation. We observed that GFRI increases corporate R&D expenditure, making enterprises innovate technology, and ultimately improving the efficiency of corporate environmental investment.

The remainder of our paper is arranged as follows.Section 2 introduces a theoretical analysis of GFRI and corporate environmental investment; Section 3 shows the empirical model and data sources; Section 4 lists the main empirical results and mechanism assessment of this paper, which confirmed hypothesis H1; Section 5 presents the results of the heterogeneity analysis; and Section 6 summarises the conclusions, policy recommendations and research deficiencies of this paper.

## 2. Policy background and research hypotheses

### 2.1 The green finance reform and innovation

Regarding monetary and financial policies, the GFRI pilot area has clarified the identification standards of green enterprises and projects, introduced green finance performance evaluation, encouraged green finance innovation, strengthened green finance supervision, and established green rating and certification institutions. In terms of fiscal policy, the GFRI pilot area sets up special financial funds and incentive mechanisms for green development to leverage financial resources, favouring green enterprises and low-carbon projects [20,28].

In addition, the pilot zones will develop green financial products tailored to local conditions. For example, Guangdong Province and Zhejiang Province have a high level of green finance development, and their business models mainly involve green credit and technology financing. The Jiangxi and Guizhou provinces have adopted green funds to help enterprises research and develop clean energy technologies and execute green projects. The Xinjiang Autonomous Region and Gansu Province have fully utilised their natural resources to develop innovative green financial products, such as "photovoltaic loans" and "wind power loans." Chongqing initiated "Yangtze River Green Financing" to improve the construction of digital infrastructure for green finance.

Green finance is mainly induced by the generation of green financial instruments and services, such as green credit, green bonds, and green funds [27]. Among them, green credit plays an increasingly important role in managing enterprises’ environmental behaviour and guiding their green technology innovation [31]. For example, Japan subsidizes and supports energy-saving technology R&D and green industries through green credit [11]. Then green credit policy significantly reduces the new financing of enterprises with high pollution and emissions and helps guide the "two high" enterprises to control pollution and reduce emissions [26]. Meanwhile, green bonds are mainly used to finance green projects and encourage enterprises to execute green projects [32]. It promotes corporate social responsibility, and investors are more inclined to invest in the project. Green funds help invest capital in projects that save energy and reduce emissions. This contributes to the progress of industrial green technology and enhances the green development ability of enterprises.

### 2.2 Research hypotheses

People began to pay attention to environmental pollution as early as the 1960s [33]. Environmental problems, such as global warming, haze, acid rain, and so on, have caused severe threats to people’s living environment and physical health [34]. It is urgent to regulate and optimize environmental problems. Thus, there was a pressing need to regulate and reduce environmental problems, and the "cost theory" of environmental investment was born.

The "cost theory" of environmental protection investment regards environmental protection investment as the cost of environmental protection activities such as pollution prevention and control. These costs often also cover the management of corporate environmental organisations and the updating of anti-pollution equipment [35]. This investment consumes the internal resources of the enterprise and increases production costs [36]. At the same time, Arouri argued that companies often localise operations in areas with low environmental standards to avoid the high environmental cleaning cost [36]. Strict environmental control leads to increased production costs for enterprises, forcing the suspension of part of their investment activities and resulting in a temporary loss in market competitiveness. After an enterprise is required to disclose environmental information and environmental protection expenditure, production costs increase in a disguised form, leading to a decrease in corporate income and value [37]. As a result, most enterprises refrain from carrying out environmental governance and investment activities subjectively [38]. A resource-based view holds that some unique resources owned by the enterprises, including specific technology, abilities, or knowledge, provide them with unique competitiveness [39,40]. To improve their competitive market advantages, enterprises must increase their investment in research and development. When the level of research and development is elevated or reaches a certain level, the investment will inevitably promote the enterprise’s product optimisation, environmental management, and environmental technology upgrades. Ultimately, the environmental investment efficiency of enterprises is improved, and the cost of pollution control is reduced [41–43].

With the introduction of the concept of sustainable development and the improvement of environmental regulations, the scope of environmental protection expenditure among enterprises is no longer limited to the "end treatment". Enterprises improve resource utilisation, produce technology, and upgrade production equipment to prevent and control pollution [44]. This process has produced economic benefits, enriching the economic sense of "investment" in environmental investment. Thus, the "investment theory" of environmental investment was born.

The Porter hypothesis holds that appropriate environmental regulations will bring innovation and application of environmental technologies to enterprises. The arrival of emerging technologies enables enterprises to simultaneously strengthen their investment efficiency in environmental protection and assume environmental responsibilities [45]. Benefits offered by the technological innovation of enterprises can even offset the costs imposed by environmental regulations, thereby generating net benefits, which is known as the innovation compensation effect. Based on Porter’s hypothesis, Jaffe and Palmer (1997) [46] studied the American manufacturing industry and found that the increase in enterprise environmental protection expenditure significantly promoted enterprise technological innovation. Ouyang et al. (2020) [47] found that environmental regulation contributes to innovation; the stronger an enterprise’s environmental protection expenditure, the stronger the effect on technological innovation, which confirmed the Porter hypothesis to some extent [47].

By combining the environmental investment theory and the purpose of the GFRI pilot policy, we can deduce that enterprises reduce costs through advanced technology and establish their corporate reputation through sustainable development in order to obtain the support of green finance, thus creating priceless assets for enterprises and improving their environmental performance [48,49]. The best choice for enterprises to obtain the support of green finance is to expand the scale and improve the efficiency of environmental investment. However, due to practical problems such as internal resource allocation, enterprises may follow the "cost theory" of environmental investment and take environmental expenditure as the production cost of enterprises [50]. Under the influence of stress, enterprises will reduce the scale of environmental protection investment to devote more limited internal resources to research and development investment. After realising green technology innovation, enterprises will improve the efficiency of environmental protection investment [51]. They may also adhere to the "investment theory" of environmental investment and the GFRI pilot policy as a kind of environmental regulation. After inclining their internal resources to the environmental investment, the innovation compensation mechanism in the "Porter hypothesis" promotes an enterprise’s technological innovation and energy saving [52,53]. Therefore, there are uncertainties in implementing the GFRI pilot policies and the scale and efficiency of investment in environmental protection. Enterprises choose to expand the scale of environmental protection investment or improve the efficiency of environmental protection investment to win the support of green finance policy. Based on this, we propose the following hypotheses:

**Hypothesis 1 (H1):** Enterprises invest limited internal resources into R&D expenditure and technological innovation, which improves the efficiency of environmental investment and reduces the scale of corporate environmental investment.
**Hypothesis 2a (H2a):** Enterprises invest limited internal resources into environmental expenditure, which expands the scale of corporate environmental investment and decreases investment efficiency.
**Hypothesis 2b (H2b):** Enterprises invest limited internal resources into environmental expenditure to expand the scale and improve the efficiency of corporate environmental investment.

## 3. Methodology

### 3.1. Data and sample selection

The data used in this study was obtained from several different sources. We collected and collated initial data on total environmental investment by listed companies from corporate social responsibility reports, sustainable development reports, environmental reports, and annual schedule annotations. Listed companies gradually started to disclose relevant environmental information after the Shenzhen and Shanghai stock exchanges issued Guidelines for Environmental Information Disclosure of Listed Companies in 2008. Considering the lag of the policy, we select 2010 as the starting point. We also collected the annual reports, financial data, R&D expenditure, and patent numbers from the China Stock Market & Accounting Research Database(CSMAR). The city, equity nature, industry, concept plate, and other data of listed enterprises were obtained from the Wind.

A-share listed enterprises that disclosed their environmental investment between 2010 and 2020 were used as research samples, and were screened as follows: (1) We excluded companies listed in 2011 and later; (2) we excluded special treatment(ST, SST, and *ST) companies; and (3) companies with more missing data were also excluded. Finally, we obtained a total of 819 company samples.

### 3.2 Research design

We constructed the following staggered DID model to assess the impact of green finance reform on enterprises’ environmental investment.

$${EPI}_{it}=\alpha_{0}+\beta_{1}{alterdid}_{rt}+\beta_{2}{Control}_{it}+{Year}_{t}+\gamma_{i}+\varepsilon_{it}$$ (1)

$$EPIE_{it}=\alpha_{0}+\beta_{1}^{’}{alterdid}_{rt}+\beta_{2}^{’}{Control}_{it}+{Year}_{t}+\gamma_{i}+\varepsilon_{it}$$ (2)

The explanatory variable is *alterdid*_*it*_, which is equal to *treat*_r_*post_*t*_. I, r, and t represent the listed enterprise, region, and time, respectively. *Treat* denotes a dummy variable in the policy pilot area. The enterprises covered by the green finance pilot policy are the treatment group, and the treat value is 1; otherwise, it is 0. *Post* refers to a dummy variable of the policy pilot time, 1 in the second year and after establishing the GFRI pilot zone; otherwise, it is 0. *Control*_*it*_ represents a group of control variables. *Year*_*t*_ denotes the time-point fixed effect. *γ*_*i*_ refers to an individual fixed effect. *ε*_*it*_ is a random error term.

### 3.3 Definitions of variables

We selected listed enterprises covered by the GFRI pilot zones as the treatment group. Listed enterprises from other regions served as the control group. The first GFRI pilot zones were established in June 2017, and Gansu Province joined in November 2019. The policy’s role has a certain lag; therefore, we regarded the second year after the establishment of the GFRI pilot area as the year in which the policy begins.

The dependent variable was the environmental protection investment (EPI) of Eq 1 and environmental investment efficiency (EPIE) of Eq 2. Drawing on the definition of Hammar et al.(2010) [54], we define EPI as funding allocated specifically for activities aimed at preventing, reducing, and eliminating pollution or any other form of environmental degradation. We referred to the research of Hammar et al. (2010) [54] and Tang et al.(2013) [55] to use "environmental expenditure/capital stock" to represent the EPI. The capital stock was the arithmetic average of the total assets at the beginning and end of the year. This measure reflects the proportion of environmental expenditure relative to the company’s overall capital. If EPI is high, it will lead to the reduction of funds for other production activities [56]; otherwise, if EPI is low, it reflects that the enterprise has not fulfilled the environmental protection task well [57]. We used the Super Slack Based Measure (super-SBM) to calculate the EPIE. The model was set as follows:

$$min\rho =\frac{1+\frac{1}{m}\sum_{i=1}^{m}S_{i}^{-}/x_{ik}}{1-\frac{1}{S}\sum_{r=1}^{S}S_{r}^{+}/y_{rk}}$$ (3)

ρ is the efficiency value. X and y are the input and output variables, respectively. M is the number of input indicators, and s is the number of output indicators. $S_{i}^{-}$ and $S_{r}^{+}$ are the amounts of relaxation in the input and output, respectively. We took enterprise environmental expenditure as the input variable and profit indicators such as return on assets(ROA), return on equity (ROE)s, and business revenue growth as the output variable.

We take into account that there may be an impact on environmental investment due to internal factors of the firm [58,59]. Following the conventional practice in the literature [60], we controlled a set of company-level characteristic variables, as shown in Eq (1), including enterprise size (Size), asset-liability ratio (Debt), return on assets (ROA), the proportion of tangible assets (FIXED), corporate investment opportunities (TobinQ), corporate cash flow (Cashflow), and enterprise expense ratio (Cost).

Moreover, green finance reform can influence enterprises’ investment in environmental protection from R&D expenditure and technological innovation. Therefore, we choose R&D expenditure and technological innovation as the mechanism variables. Technological innovation is represented by the number of patents of enterprises. The definitions of all these variables are provided in Table 1.

**Table 1:** Variable definitions.

Category	Variable	Definitions
Dependent variable	EPI	Environmental expenditure/capital stock
	EPIE	Calculation of the SBM model
Explanatory variable	alterdid	treat×post
Control variable	Size	We took the natural log of the total assets of the firm
	Debt	The ratio of total liabilities to total assets
	ROA	The ratio of net profit after tax to total assets
	FIXED	The ratio of net fixed assets to total assets
	TobinQ	(Total market value + book value of liabilities)/total assets
	Cashflow	The ratio of net cash flow to ending current liabilities
	Cost	The ratio of total expenses to total revenues
Mechanism variable	Lninve	The natural logarithm of R&D input was taken
	Patent	Number of enterprise invention patents at the end of the year

## 4. Empirical results

### 4.1 Descriptive statistics

Table 2 provides the descriptive statistics of the main variables. The maximum EPI was 0.455, while the minimum was 0.0001. The maximum EPIE was 1.26, and the minimum was 0.05. These results indicate that the scale and efficiency of environmental investment vary greatly among enterprises.

**Table 2:** Descriptive statistics.

Variable	N	Mean	Std dev	Min	Max
EPI	9009	1.133	2.850	0.0001	45.51
EPIE	9009	0.100	0.040	0.050	1.260
Lninve	7465	17.80	1.920	4.740	24.10
Patent	4198	2.400	13.93	0	579.0
Size	9009	22.58	1.400	17.53	28.64
Debt	9009	0.53	0.250	0.0100	2.729
ROA	9009	0.03	0.0900	-1.280	4.490
Cost	9009	0.002	0.0071	-0.084	0.446
Cashflow	9009	0.04	0.0900	-4.270	0.600
FIXED	9009	0.28	0.180	0.0003	0.950
TobinQ	9009	1.930	1.850	0.690	56.66

### 4.2. Baseline results

Table 3 reports the regression results of the staggered DID. Columns (1) and (3) illustrate that the results are consistent with the baseline regression in the absence of control variables. Column (2) reports that the *alterdid* coefficient is significantly negative at the 1% level, indicating that the establishment of the GFRI pilot zone had a significant inhibitory effect on the scale of corporate environmental investment in the pilot zone. Specifically, GFRI can significantly reduce the scale of corporate environmental investment by about 0.82. Column (4) shows that the *alterdid* coefficient is positive and significant at the 1% level, indicating that enterprises in the GFRI experiment zone have improved the efficiency of corporate environmental investment. GFRI can significantly increase the environmental investment efficiency of enterprises by about 0.48%. Based on the above empirical results, most enterprises in the pilot zone improved the efficiency of their corporate environmental investment and simultaneously reduced the scale of their corporate environmental investment after the establishment of the GFRI pilot zone.

**Table 3:** Green finance reform and enterprise environmental protection investment.

	(1)	(2)	(3)	(4)
Variable	EPI		EPIE	
alterdid	-0.833***	-0.822***	0.00481***	0.00477***
	(0.115)	(0.115)	(0.00121)	(0.00121)
Size		-0.324***		0.00147**
		(0.0554)		(0.000582)
Debt		0.834***		0.00245
		(0.152)		(0.00182)
ROA		0.561**		-0.00981**
		(0.277)		(0.00291)
Cost		-0.0131		0.000137
		(0.0363)		(0.000381)
Cashflow		-0.700**		-0.00510*
		(0.303)		(0.00319)
FIXED		-0.941**		0.00411
		(0.271)		(0.00284)
TobinQ		-0.0363*		-0.000071
		(0.0197)		(0.000207)
Individual FE	Yes	Yes	Yes	Yes
Year FE	Yes	Yes	Yes	Yes
Observations	9,009	9,009	9,009	9,009
R-squared	0.488	0.492	0.649	0.651

*** p<0.01

** p<0.05

* p<0.1.

Based on a natural resource-based view, the increase in enterprise R&D investment will increase the environmental performance of enterprises. Bostian et al. (2016) [61] also believed that increased R&D investment provides new technologies, which would reduce energy use and pollution emissions. However, according to the "cost theory" of environmental investment, the internal resources of enterprises are limited, and the environmental investment of enterprises will be forced to decrease following increased R&D investment. The empirical results in Table 3 verify this. The following paper will apply the mechanism test to determine whether GFRI improves enterprises’ R&D expenditure and technological innovation capability.

### 4.3. Underlying mechanisms

We aim to explore the impact mechanism of pilot green finance policies on micro-enterprises’ environmental investment. We selected R&D expenditure and enterprise technological innovation as variables to determine the micro-effect mechanism of green finance pilot policies.

According to the results of columns (1) and (2) in Table 4, the coefficient of alterdid is significantly positive at the level of 10%. We found that after the green finance reform, the R&D expenditure of enterprises increased and the number of patents obtained by enterprises increased significantly. Combined with the results of Table 3, we observed that after the establishment of the GFRI pilot area, enterprises’ R&D investment increased and their innovation ability improved, significantly enhancing their environmental protection investment efficiency. So, enterprises can conserve part of their environmental protection expenditure for innovation to form a virtuous circle of green finance and enterprise environmental protection investment. Therefore, H1 is satisfied, while H2a and H2b are rejected.

**Table 4:** Green finance reform and enterprise R&D/technological innovation.

	(1)	(2)
Variable	Lninve	Patent
alterdid	0.0906*	1.921**
	(0.0524)	(0.974)
Individual FE	Yes	Yes
Year FE	Yes	Yes
Control	Yes	Yes
Observations	7,465	4,198
R-squared	0.804	0.684

Furthermore, we divide enterprises into low R&D and high R&D based on pre-period values, so as to more intuitively observe the relationship between R&D expenditure and efficiency. According to the results in Table 5, it can be found that the reduction in EPI of high R&D companies is larger, but the corresponding increase in EPIE is larger. This indicates that high R&D firms are able to achieve efficiency gains. Conversely, low R&D firms do not experience the same efficiency gains as high R&D firms.

**Table 5:** EPI/EPIE for high R&D/low R&D firms.

	(1)	(2)	(3)	(4)
	High R&D firms		Low R&D firms	
VARIABLES	EPI	EPIE	EPI	EPIE
alterdid	-0.965***	0.0063***	-0.440**	0.0040
	(0.162)	(0.00191)	(0.214)	(0.00252)
Individual FE	Yes	Yes	Yes	Yes
Year FE	Yes	Yes	Yes	Yes
Control	Yes	Yes	Yes	Yes
Observations	3,598	3,598	3,807	3,807
R-squared	0.563	0.675	0.593	0.668

**Fig 1:**
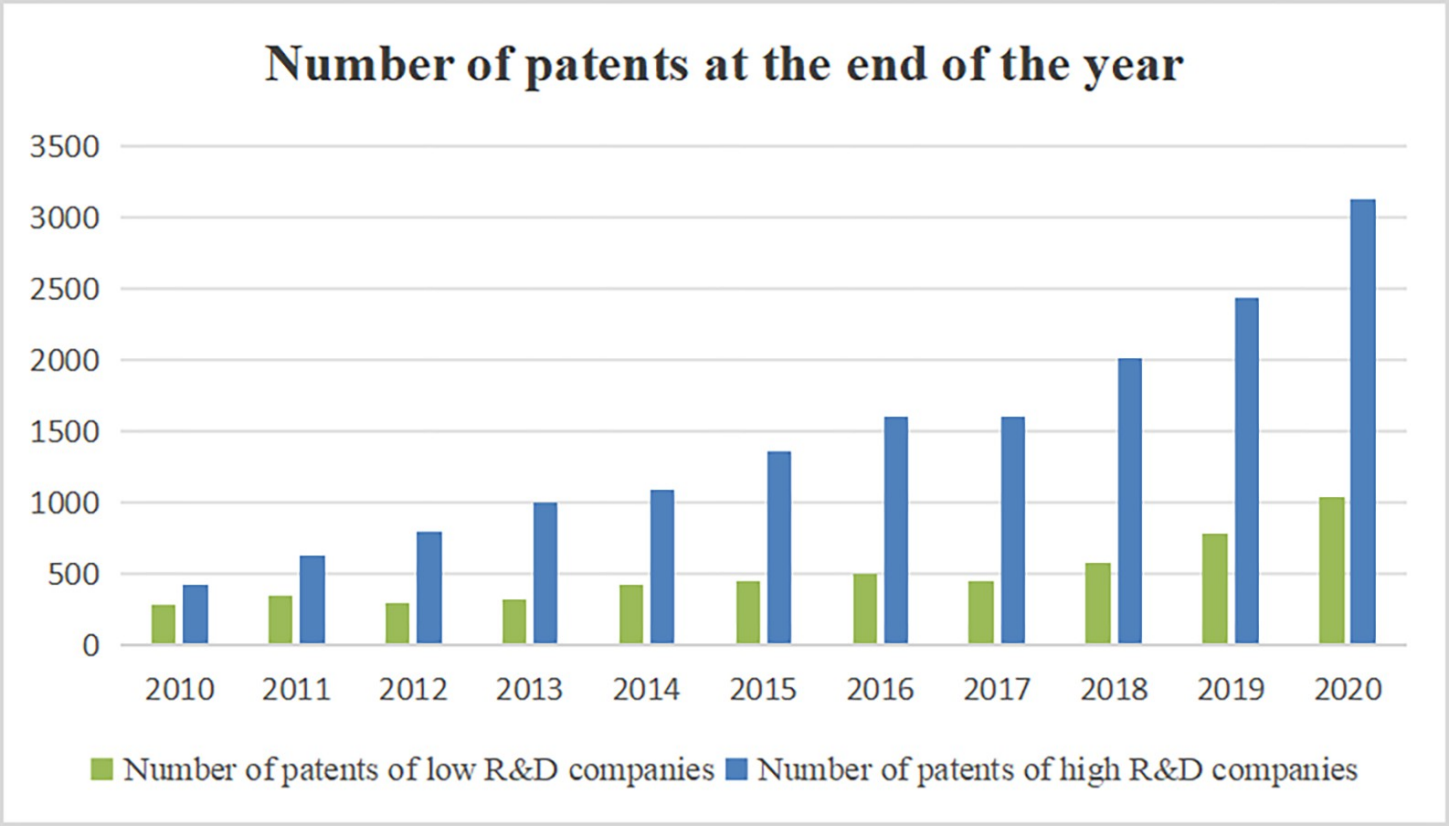
Number of invention patents of enterprises at the end of the year.

In addition, we counted the number of invention patents of low-R&D and high-R&D enterprises to draw the graph. It can be seen from Fig 1 that there is no significant change in the number of invention patents of low-R&D companies before 2017, but there is a linear relationship after 2017. The number of invention patents of high-R&D companies increased by less than 300 per year before 2017, but by more than 500 per year after 2017. Therefore, this also supports the fact that the innovation and efficiency of high R&D companies are improving.

### 4.4 Parallel trends test

**Fig 2:**
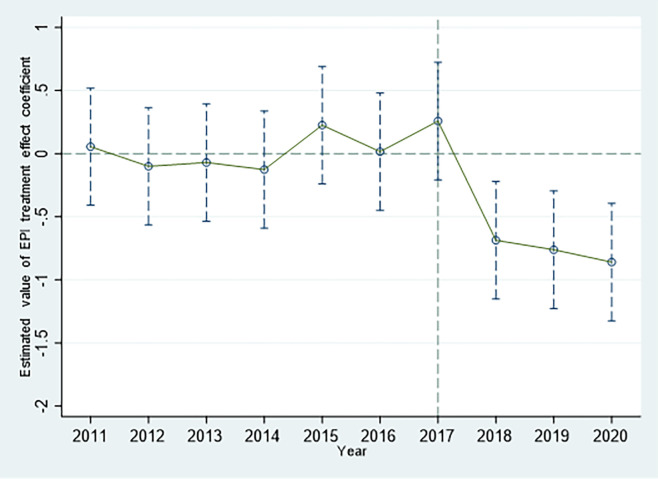
Parallel trend test of corporate environmental investment scale.

**Fig 3:**
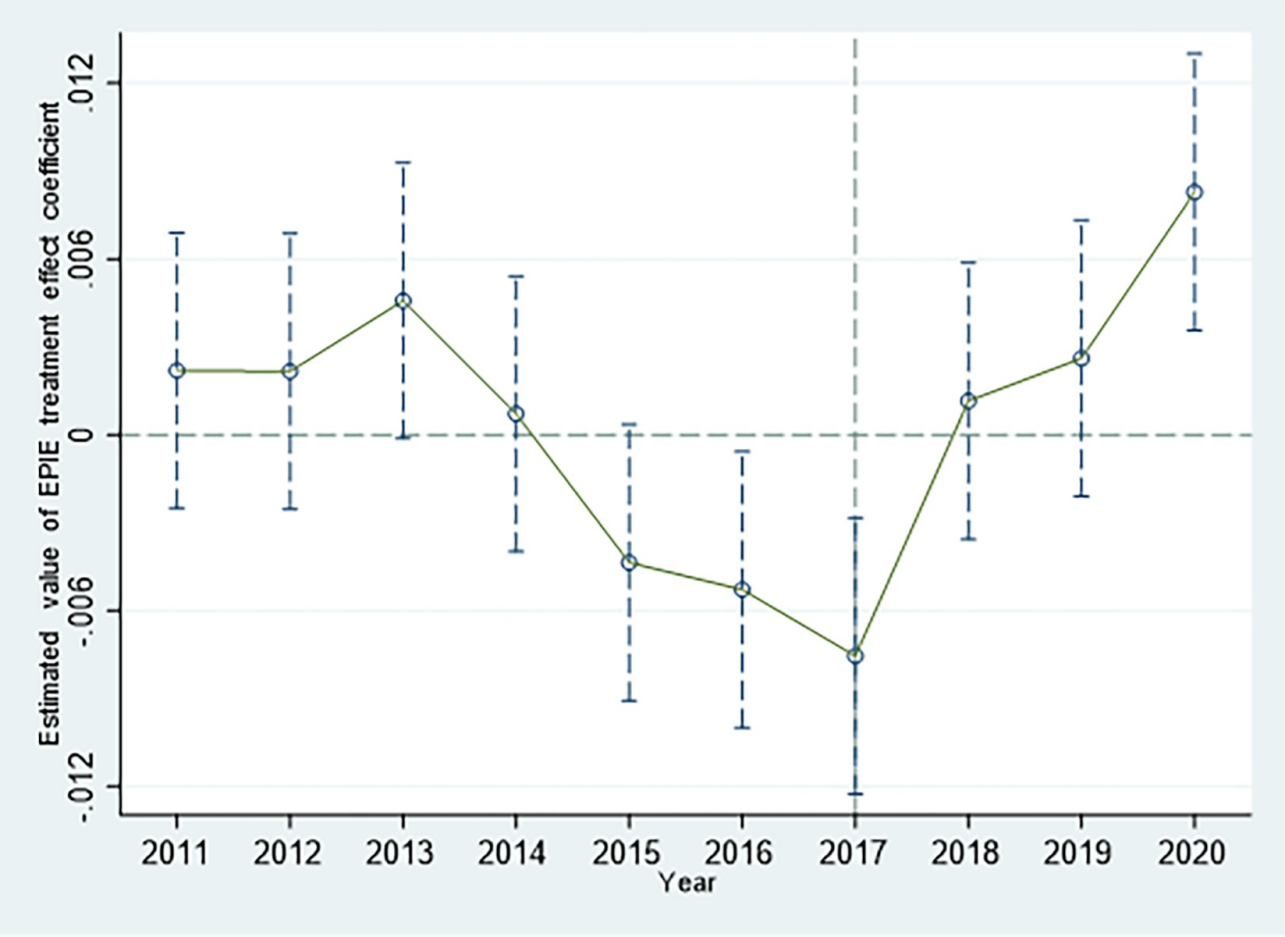
Parallel trend test of corporate environmental investment efficiency.

Fig 2 demonstrates that the estimated results of the EPI treatment effects before 2017 were all around the ordinate 0 points, and there was a significant fluctuation in 2017, when the policy was implemented. After 2017, the estimated results of the treatment effect deviated significantly from 0 points on the ordinate axis and the fluctuation trend before 2017. Fig 3 shows the rapid deviation from below-axis 0 to above-axis 0 in the estimated results of the EPIE treatment effect after 2017 which represents a significant improvement in the environmental investment efficiency in the treatment group. In other words, after 2017, the change trends in pilot and non-pilot areas began to show significant differences, with corporate environmental investment scale declining significantly in the year the pilot area was established (Fig 2), while the corporate environmental investment efficiency increased significantly (Fig 3). Therefore, the hypothesis of a parallel trend between the scale and efficiency of corporate environmental investment can be graphically verified.

### 4.5 Robustness checks

#### 4.5.1. PSM-DID

We used the PSM-DID model to test the impact of green finance reform on enterprises’ environmental investment. For the convenience of comparison, the same control variables and fixed effects as above were selected. We utilised the nearest neighbour matching method to match the treatment group with the control group to reduce the endogenous problems caused by self-selection bias in establishing the GFRI pilot zone. The specific results are shown in Table 6. The estimated results, correlation, and significance level are consistent with the baseline regression results in Table 3.

**Table 6:** The PSM-DID regression results.

	(1)	(2)	(3)	(4)
Variable	EPI		EPIE	
alterdid	-0.828***	-0.830***	0.00625***	0.00627***
	(0.183)	(0.183)	(0.00155)	(0.00156)
Individual FE	Yes	Yes	Yes	Yes
Year FE	Yes	Yes	Yes	Yes
Control	No	Yes	No	Yes
Observations	3,810	3,810	3,810	3,810
R-squared	0.600	0.603	0.741	0.742

#### 4.5.2 Alternative measures of environmental investment

We used the natural logarithm of environmental investment (lnexp) instead of the explained variable (EPI) and found that the green finance reform still reduced enterprises’ investment in environmental protection. The regression results are shown in Table 7. They are basically consistent with the regression results in Table 3, which proves the robustness of the above conclusions.

**Table 7:** Alternative measures of environmental investment.

	(1)	(2)	(3)
Variable	lnexp	lnexp	lnexp
alterdid	-0.668***	-0.451***	-0.690***
	(0.0643)	(0.0803)	(0.0614)
Individual FE	Yes	No	Yes
Year FE	Yes	No	Yes
Control	No	Yes	Yes
Observations	9,009	9,009	9,009
R-squared	0.765	0.320	0.787

#### 4.5.3 Shorten the time window

We excluded the sample data of the first and last two years to re-estimate. As shown in Table 8, the results were consistent with the conclusion obtained from the regression results in Table 3, proving that the above conclusion is robust.

**Table 8:** The baseline result of shortening the time window.

	(1)	(2)	(3)	(4)
Variable	EPI		EPIE	
alterdid	-0.789***	-0.773***	0.00357**	0.00338**
	(0.143)	(0.143)	(0.00146)	(0.00146)
Individual FE	Yes	Yes	Yes	Yes
Year FE	Yes	Yes	Yes	Yes
Control	No	Yes	No	Yes
Observations	7,470	7,470	7,470	7,470
R-squared	0.519	0.522	0.650	0.653

### 4.6 Placebo test

**Fig 4:**
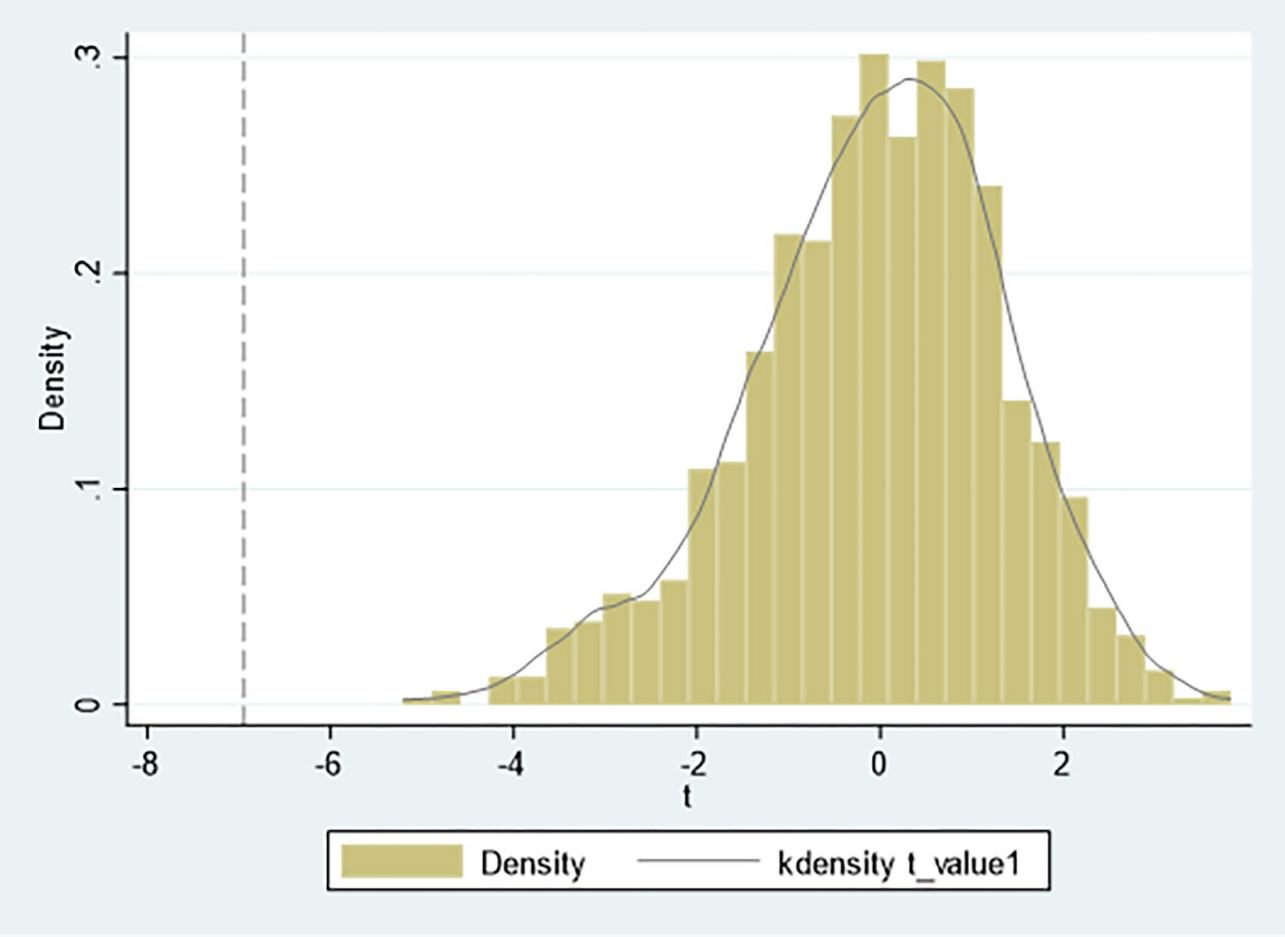
Placebo test for EPI.

**Fig 5:**
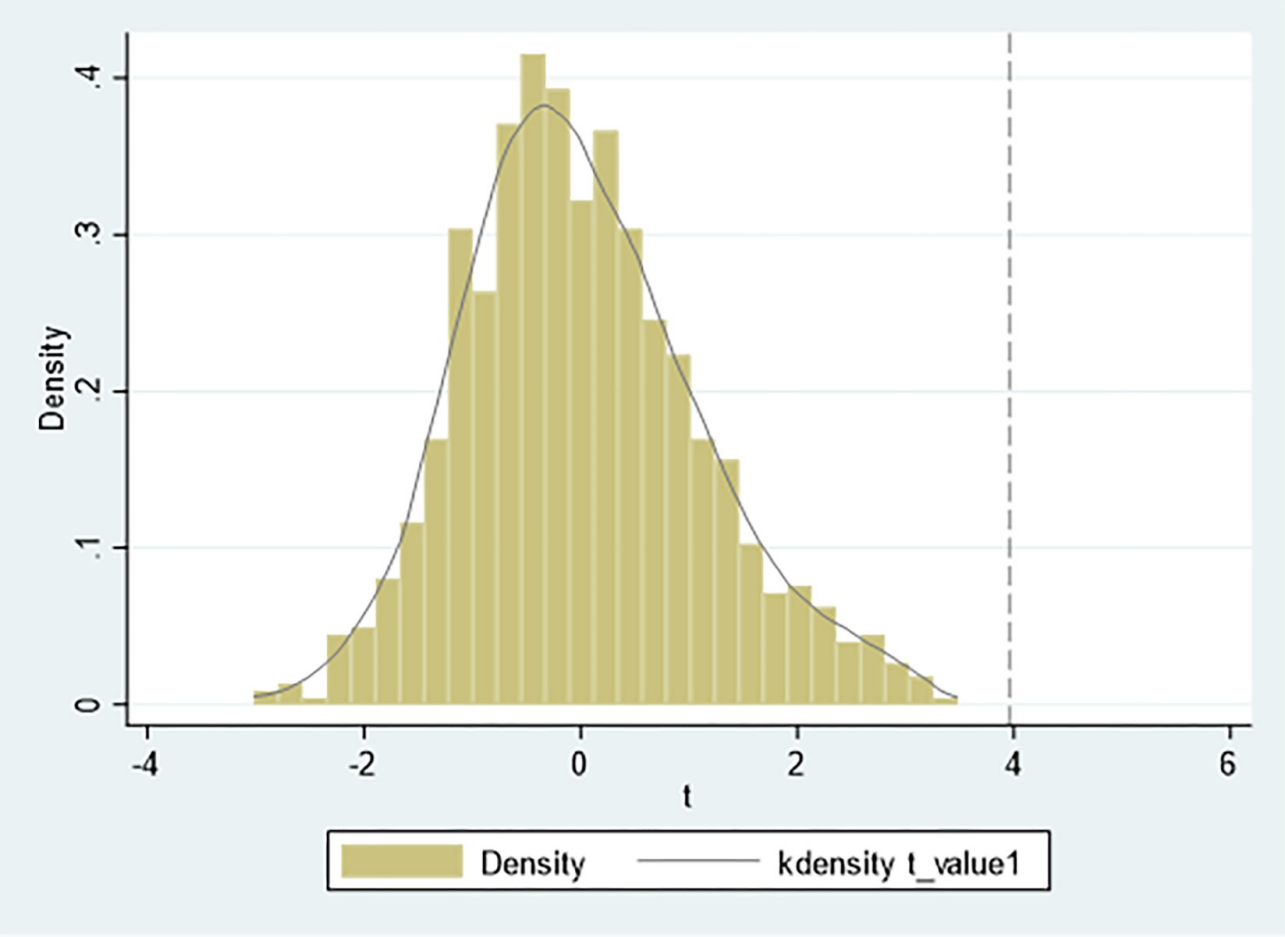
Placebo test for EPIE.

In order to further confirm that this result is caused by the impact of the green finance pilot zone policies rather than other random factors, this paper adopts the placebo test method. The idea behind this method is to randomly generate the treatment groups and re-run the DID regression. In recent years, this method has been widely used in the DID research, such as La Ferrara et al.(2012) [62], Liu and Lu(2015) [63]. We repeatedly generated 1000 random treatment groups. The true t-statistics in Figs 4 and 5 are -6.95 and 3.96, respectively. The t-statistics in the regression results are different from the true values. Therefore, this paper passes the placebo test.

## 5. Further tests

### 5.1. Heterogeneity effects

Drawing on the findings of Pan et al. (2019) [64], we defined enterprises in 15 heavily polluting industries as polluting enterprises. According to the concept classification of listed companies in the Wind, we defined the enterprises in 31 conceptual sectors, such as green power and environmental protection governance, as green enterprises.

From columns (1) and (3) of Table 9, we observed that the interaction term coefficients of green and polluting enterprises are negative. The absolute value of the interaction term coefficient of green enterprises is large and significant at a 1% level, while that of polluting enterprises is significant at a 10% level. The results demonstrated that the decrease in green enterprises’ environmental investment scale was markedly more significant than that of polluting enterprises after implementing the GFRI pilot policy. However, we observed from columns (2) and (4) that the GFRI does not significantly affect the efficiency of the corporate environmental investment of green enterprises, but helps polluting enterprises to improve efficiency of their corporate environmental investment.

**Table 9:** Industry type heterogeneity.

	(1)	(2)	(3)	(4)
	green enterprises		polluting enterprises	
Variable	EPI	EPIE	EPI	EPIE
alterdid	-1.445***	0.00191	-0.449**	0.00475**	
	(0.276)	(0.00282)	(0.203)	(0.00193)	
Individual FE	Yes	Yes	Yes	Yes	
Year FE	Yes	Yes	Yes	Yes	
Control	Yes	Yes	Yes	Yes	
Observations	1,050	1,050	1,563	1,563	
R-squared	0.617	0.623	0.378	0.698	

We classified the samples according to enterprise ownership and obtained two types of state-owned and non-state-owned enterprises. The samples were substituted into Eqs (1) and (2) for calculation, and the specific estimation results are shown in Table 10. Columns (1) and (3) show that green finance reform significantly reduces the scale of enterprise environmental protection investment of state-owned and non-state-owned enterprises, and the scale of environmental protection investment of non-state-owned enterprises decreases even more. The results in columns (2) and (4) showed that after the GFRI, the improvement of environmental protection investment efficiency of state-owned enterprises was more significant than that of non-state-owned enterprises.

**Table 10:** Heterogeneity of enterprise ownership.

	(1)	(2)	(3)	(4)
	State-owned enterprise		Non-state-owned enterprise	
Variable	EPI	EPIE	EPI	EPIE
alterdid	-0.350**	0.00570***	-1.075***	0.00415***
	(0.181)	(0.00223)	(0.150)	(0.00092)
Individual FE	Yes	Yes	Yes	Yes
Year FE	Yes	Yes	Yes	Yes
Control	Yes	Yes	Yes	Yes
Observations	5,057	5,057	3,927	3,927
R-squared	0.507	0.618	0.536	0.801

A possible reason is that state-owned enterprises generally shoulder greater social and environmental responsibility. However, non-state-owned enterprises are mostly foreign-funded enterprises and small, medium, and micro enterprises (MSMEs). Foreign-funded enterprises and MSMEs are effective at introducing green innovation technologies from abroad, reducing corporate environmental protection expenditures, and converting excess funds into R&D expenditures, technology imports, etc.

## 6. Conclusion

### 6.1 Conclusion

This study discussed the impact of establishing the GFRI pilot area on the environmental investment of local enterprises. We found that GFRI improves the efficiency of corporate environmental investment but reduces its scale. After selecting different matching methods, replacing dependent variables, and shortening the time window, this conclusion remained robust. Further investigation showed that GFRI increased the efficiency of corporate environmental investment by increasing the R&D investment and the number of invention patents. This study provides evidence that the implementation of GFRI in China is conducive to sustainable development and the green development of enterprises.

### 6.2 Policy implications

First, the key tasks, supporting policies, and financial products of the GFRI pilot zone should focus more on helping enterprises to innovate in green technology. The results of this paper demonstrated that enterprises were able to reduce their environmental protection investment and increase R&D investment after the establishment of the GFRI pilot zone, thus facilitating their own green technology innovation. This was due to the improvement of environmental investment efficiency. Therefore, the government should give full play to green financial tools, such as stimulating the innovation vitality of enterprises by means of special fund support and tax incentives, and strengthening the supervision of the implementation of green finance, so as to ensure that the new funds are used for technological innovation and R&D investment and improve the policy effect. This will help leverage financial resources to favour green technology enterprises and low-carbon projects, and ultimately improve the ecological environment and reduce corporate carbon emissions while realizing corporate technological innovation.

Second, the government should continue to deepen the supply-side structural finance reform, establish a long-term mechanism to support the innovative development of green finance, and provide related financial products to various types of enterprises to promote their green transformation and development. This paper concludes that reducing the scale of environmental protection investment and increasing the R&D investment of polluting enterprises does not play a significant role in promoting the efficiency of environmental protection investment. Thus, it is more necessary for them to increase the scale of environmental protection investment, update pollution control equipment, introduce advanced environmental protection technology, and reduce environmental pollution at the "pollution control end". Specifically, the government can formulate differentiated policy measures according to the different characteristics of green enterprises and polluting enterprises. Future policy making should pay more attention to the green transformation of polluting enterprises so that they can play a more active role in green finance development.

Finally, there are some shortcomings in this paper that should be noted. Since Chongqing joined the GFRI pilot zone in 2022, the treatment group in this study does not include Chongqing’s listed companies. In addition, the distinction between green enterprises and polluting enterprises in this paper can be further supplemented in future research. Moreover, there are still some listed enterprises in China that have not disclosed environmental information. It is difficult for us to collect the environmental performance of non-disclosing firms. In the heterogeneity analysis, differences in the impact of GFRI on corporate environmental investment should also be considered due to variations in the degree of marketisation and the level of green finance development.

## Supporting information

S1 Raw data (RAR)
